# Hepatitis B Virus Disrupts Mitochondrial Dynamics: Induces Fission and Mitophagy to Attenuate Apoptosis

**DOI:** 10.1371/journal.ppat.1003722

**Published:** 2013-12-05

**Authors:** Seong-Jun Kim, Mohsin Khan, Jun Quan, Andreas Till, Suresh Subramani, Aleem Siddiqui

**Affiliations:** 1 Division of Infectious Diseases, Department of Medicine, University of California, San Diego, La Jolla, California, United States of America; 2 Section of Molecular Biology, Division of Biological Sciences, University of California, San Diego, La Jolla, California, United States of America; 3 San Diego Center for Systems Biology, University of California, San Diego, La Jolla, California, United States of America; University of Alabama at Birmingham, United States of America

## Abstract

Human hepatitis B virus (HBV) causes chronic hepatitis and is associated with the development of hepatocellular carcinoma. HBV infection alters mitochondrial metabolism. The selective removal of damaged mitochondria is essential for the maintenance of mitochondrial and cellular homeostasis. Here, we report that HBV shifts the balance of mitochondrial dynamics toward fission and mitophagy to attenuate the virus-induced apoptosis. HBV induced perinuclear clustering of mitochondria and triggered mitochondrial translocation of the dynamin-related protein (Drp1) by stimulating its phosphorylation at Ser616, leading to mitochondrial fission. HBV also stimulated the gene expression of Parkin, PINK1, and LC3B and induced Parkin recruitment to the mitochondria. Upon translocation to mitochondria, Parkin, an E3 ubiquitin ligase, underwent self-ubiquitination and facilitated the ubiquitination and degradation of its substrate Mitofusin 2 (Mfn2), a mediator of mitochondrial fusion. In addition to conventional immunofluorescence, a sensitive dual fluorescence reporter expressing mito-mRFP-EGFP fused in-frame to a mitochondrial targeting sequence was employed to observe the completion of the mitophagic process by delivery of the engulfed mitochondria to lysosomes for degradation. Furthermore, we demonstrate that viral HBx protein plays a central role in promoting aberrant mitochondrial dynamics either when expressed alone or in the context of viral genome. Perturbing mitophagy by silencing Parkin led to enhanced apoptotic signaling, suggesting that HBV-induced mitochondrial fission and mitophagy promote cell survival and possibly viral persistence. Altered mitochondrial dynamics associated with HBV infection may contribute to mitochondrial injury and liver disease pathogenesis.

## Author Summary

Hepatitis B virus (HBV) chronic infections represent the common cause for the development of hepatocellular carcinoma. Mitochondrial liver injury has been long recognized as one of the consequences of HBV infection during chronic hepatitis. Mitochondria are dynamic organelles that undergo fission, fusion, and selective-autophagic removal (mitophagy), in their pursuit to maintain mitochondrial homeostasis and meet cellular energy requirements. The clearance of damaged mitochondria is essential for the maintenance of mitochondrial and cellular homeostasis. We observed that HBV and its encoded HBx protein promoted mitochondrial fragmentation (fission) and mitophagy. HBV/HBx induced the expression and Ser616 phosphorylation of dynamin-related protein 1 (Drp1) and its subsequent translocation to the mitochondria, resulting in enhanced mitochondrial fragmentation. HBV also promoted the mitochondrial translocation of Parkin, a cytosolic E3 ubiquitin ligase, and subsequent mitophagy. Perturbation of mitophagy in HBV-infected cells resulted in enhanced mitochondrial apoptotic signaling. This shift of the mitochondrial dynamics towards enhanced fission and mitophagy is essential for the clearance of damaged mitochondria and serves to prevent apoptotic cell death of HBV-infected cells to facilitate persistent infection.

## Introduction

Hepatitis B virus (HBV) infection affects nearly 350 million people worldwide and leads to chronic liver disease, liver failure, and hepatocellular carcinoma (HCC) [1], [2]. HBV is an enveloped DNA virus that belongs to the *Hepadnavirus* family. HBV DNA genome encodes four overlapping open reading frames designated as pre-S/S (the hepatitis B surface antigen, HBsAg), C (core/e antigen, HBc/eAg), P (polymerase, reverse transcriptase), and X (HBx) [1], [2]. HBx is a regulatory protein with multiple functions involved in various cellular and physiological processes including a key role in the maintenance of viral replication [3]. It is predominantly localized to the cytoplasm and also associates with mitochondria via its interaction with voltage-dependent anion-selective channel 3 (VDAC3) [4]–[7]. This association leads to a decrease in mitochondrial transmembrane potential (ΔΨm) and depolarization of mitochondria [3], [4], [8]. HBx also participates in activating transcription of whole host of cellular genes via protein-protein interactions both in the nucleus and cytoplasm [3], [7], [9]–[11]. HBx is not directly oncogenic but participates substantially in the process of liver oncogenesis [9], [12]. HBx is a regulatory protein with pleiotropic activities and has been shown to promote endoplasmic reticulum (ER) stress, oxidative stress, deregulation of cellular calcium homeostasis, and mitochondrial dysfunction [3]. HBx also modulates the activation of several latent transcription factors such as nuclear factor-kappa B (NF-κB), signal transducer and activator of transcription 3 (STAT-3), with resultant activation of cytoprotective genes [8], [13]. The multiple effects of HBx protein may be a consequence of the trigger of the ER-mitochondria-nuclear nexus of signal transduction pathways.

Mitochondrial injury and oxidative stress are prominent features of chronic Hepatitis B and C [14], [15]. Histological manifestation of swollen mitochondria and mitochondria lacking cristae directly implicates mitochondrial injury in HBV-associated liver disease pathogenesis. HBV infection is associated with deregulated cellular Ca^2+^ signaling, mitochondrial depolarization and dysfunction and reactive oxygen species (ROS) generation [3],[8],[16]. HBV-induced elevated cellular ROS levels can also promote mitochondrial dysfunction [15]. Dysfunctional or damaged mitochondria trigger a vicious cycle of mitochondrial damage and ROS generation, which is detrimental for cell survival and can be confounded by rapid turnover of damaged mitochondria [17]. The removal of dysfunctional mitochondria is orchestrated by asymmetric mitochondrial fission to eliminate the damaged mitochondria by subsequent mitophagy (selective autophagy of mitochondria) [18]. Mitochondria subjected to physiological stress usually undergo perinuclear clustering, which precedes both mitochondrial fission and mitophagy [19].

HBV and in particular, HBx have been shown to induce bulk autophagy [20]–[24]. In this study, we investigated HBV-induced aberrant mitochondria dynamics, and mitophagy. Our data revealed that HBV shifts the balance of mitochondrial dynamics towards enhanced fission and promotes selective autophagic degradation of damaged mitochondria via mitophagy. HBV triggered mitochondrial fission by promoting mitochondrial translocation of Drp1 via upregulation of Drp1 Ser616 phosphorylation. HBV upregulated proteins that mediate mitophagy and induced the elimination of dysfunctional mitochondria via mitophagy. More specifically, mitochondrial translocation of Parkin a cytosolic E3 ubiquitin ligase was observed in HBV/HBx expressing cells resulting in its self-ubiquitination and of its substrate, mitofusin 2 (Mfn2). In addition to confocal microscopy, using a novel dual fluorescence reporter Mito-mRFP-EGFP, we demonstrated that HBV/HBx induces complete mitophagy evident by fusion of mitophagosome with lysosome. Our studies also showed that HBx protein alone or in the context of HBV full genome, is a critical activator of HBV-induced aberrant mitochondrial dynamics. Further, we demonstrated that inhibition of mitophagy by silencing Parkin results in enhanced mitochondrial apoptotic signaling in HBV-infected cells, suggesting that induction of mitochondrial fission and subsequent mitophagy subvert apoptosis impending due to accrued mitochondrial injury in HBV-infected cells. In summary, our results suggest that HBV-mediated modulation of mitochondrial dynamics may promote cell viability of infected cells. We envisage that the altered mitochondrial dynamics and induction of mitophagy possibly contribute to the persistence of HBV-infected hepatocytes. However a careful and rigorous examination of HBV-induced mitochondrial regulation and its relevance to persistent phenotype of infected hepatocytes is required to confirm this finding in *in vivo* conditions.

## Results

### HBV/HBx induces Drp1 translocation to mitochondria and mitochondrial fragmentation

**Figure 1:**
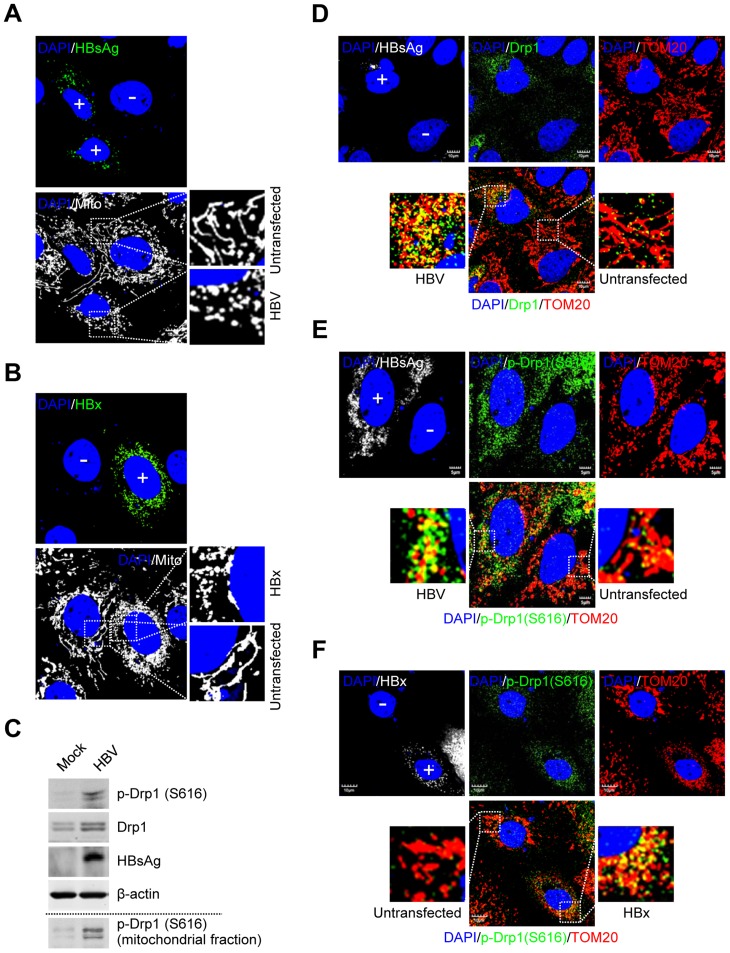
HBV/HBx promotes mitochondrial fission via Drp1 stimulation and activation. (**A** and **B**) Confocal microscopy images showing mitochondrial fragmentation in HBV- and HBx-expressing cells, respectively. Huh7 cells were transiently transfected with HBV (**A**) or the HBx-flag construct (**B**). At 2 days post-transfection, cells prestained with MitoTracker (Mito, white) were immunostained with anti-HBsAg (**A**, green) and anti-flag (**B**, green) antibodies, respectively. In the zoomed images, typical tubular mitochondria in untransfected cells and fragmented mitochondria in transfected cells are shown. (**C**) Whole cell lysates extracted from Huh7 cells with empty vector (Mock) and those with HBV construct were analyzed by Western blotting with antibodies against the indicated proteins. β-actin was used as an internal loading control. Mitochondrial fraction (bottom panel) isolated from HepAD38 cells (see the purity in **Figure 2B**) was analyzed by Western blotting with phospho-Drp1 (Ser616) antibody. (**D–F**) Confocal immunofluorescence showing mitochondrial translocation of Drp1 in HBV- and HBx-expressing cells, respectively. Huh7 cells transfected with HBV (**D** and **E**) or HBx-flag construct (**F**) were immunostained with antibodies specific to TOM20 (red), Drp1 (**D**, green), and phospho-Drp1 (Ser616) (**E** and **F**, green), respectively. HBV and HBx gene expression (white) was verified using anti-HBsAg (**D** and **E**) and anti-flag (**F**) antibodies, respectively. In the zoomed images, the yellow color indicates the merge of Drp1 or phospho-Drp1 (Ser616) with mitochondria. (**A**, **B**, **D**, **E**, and **F**) Transfected (+) and untransfected (−) cells are marked. Nuclei were stained with DAPI (blue).

We investigated the HBV-induced morphological changes of mitochondria in the human hepatoma Huh7 cells transiently expressing wild-type 1.3mer HBV genome (hereafter referred to as HBV). As shown in Figure 1A, distinct fragmented mitochondrial morphology was observed in HBV-expressing cells, compared to the typical tubular mitochondria in untransfected cells. HBx-expressing cells also displayed similar mitochondrial fragmentation (Figure 1B). HBV/HBx-expressing cells displayed prominent mitochondrial clustering in the perinuclear regions (Figure 1A and 1B), consistent with a previous report [19]. We then determined whether HBV infection triggered Drp1-mediated mitochondrial fission. As shown in Figure 1C, HBV stimulated both the expression and phosphorylation (Ser616) of Drp1. Mitochondrial translocation of Drp1 is modulated by phosphorylation at Ser616 by cyclin B/cyclin-dependent kinase 1 (Cdk1) [25]. HBV gene expression has been shown to stimulate cyclin B/Cdk1 [26]–[30]. Similar results were obtained in HBx-expressing cells (Figure S1C). Next, we examined if HBV induces Drp1 translocation to mitochondria by confocal microscopy. HBV-expressing cells displayed enhanced mitochondrial translocation of Drp1, compared to uninfected cells. (Figure 1D, see merged yellow spots). Using Drp1 antibody that specifically recognizes phosphorylated Ser616 residue, we demonstrated that most of Drp1 recruited to the mitochondria in HBV-infected cells is phosphorylated at Ser616 residue (Figure 1E). Similar results were obtained in HBx-expressing cells (Figure 1F). In support of these results, we demonstrated the accumulation of phosphorylated Drp1 in purified mitochondrial fraction from HepAD38 cells that stably express whole HBV genome under tetracycline-repressible promoter (Figure 1C, bottom panel) [31]. Together, these results indicate that HBV/HBx promotes mitochondrial fission via Drp1 translocation.

### HBV/HBx induces Parkin translocation to mitochondria

**Figure 2:**
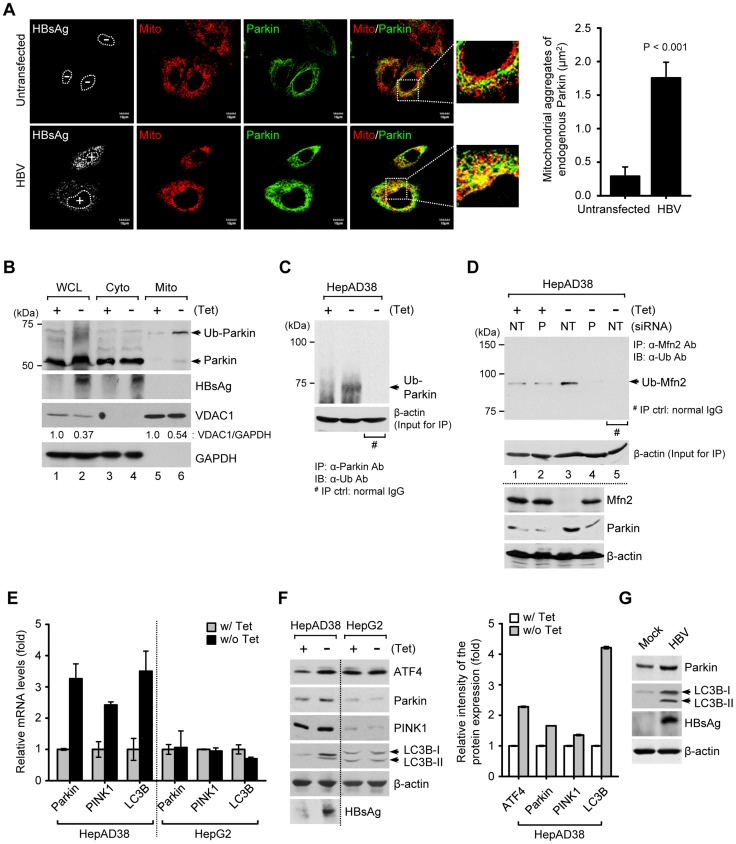
HBV induces mitochondrial translocation of Parkin and stimulates mitophagy-related genes. (**A**) Huh7 cells transfected with HBV construct were prestained with MitoTracker (Mito, red) and subsequently, immunostained with anti-Parkin (green) and anti-HBsAg (white) antibodies. Nuclei are demarcated with white dots. Transfected (+) and untransfected (−) cells are marked. In the zoomed images, yellow color indicates the accumulation of endogenous Parkin recruited to the mitochondria. Quantification of fluorescence intensity of Parkin aggregates on the mitochondria in HBV-expressing cells. (**B**) The cytosolic (Cyto) and mitochondrial (Mito) fractions were isolated from HepAD38 cells grown in the absence or presence of tetracycline for 48 h. Cellular fractions were analyzed by Western blotting with antibodies specific for the indicated proteins. Fractions: WCL, whole cell lysates; Cyto, purified cytoplasm; Mito, purified mitochondria. Organelle markers: VDAC1, mitochondria; GAPDH, cytoplasm. (**C**) Parkin protein in whole cell lysates of HBV-expressing cells was immunoprecipitated by anti-Parkin antibody, followed by immunoblotting (IB) with anti-ubiquitin (Ub) antibody. Normal rabbit IgG was used as a control for immunoprecipitation (IP). Western blot analysis for β-actin indicates equivalent amount of cell lysates for IP (**C** and **D**). (**D**) Mfn2 protein in whole cell lysates extracted from HepAD38 cells transfected with non-targeting (NT) and Parkin (P) siRNA, respectively, for 48 h was immunoprecipitated by anti-Mfn2 antibody, followed by immunoblotting (IB) with anti-ubiquitin (Ub) antibody. Normal mouse IgG was used as a control for immunoprecipitation (IP). The protein expression was analyzed by Western blotting with antibodies specific to Mfn2, Parkin, and β-actin proteins. (**E** and **F**) HepAD38 and HepG2 cells were grown in the absence or presence of tetracycline (1 µg/ml) for 12 h. (**E**) Intracellular mRNA levels of Parkin, PINK1, and LC3B were analyzed by real-time qRT-PCR. GAPDH was used to normalize changes in Parkin, PINK1, and LC3B gene expression. (**F**) The protein expression was analyzed by Western blotting with antibodies specific for the indicated proteins. β-actin was used as a loading control (**F** and **G**). The relative intensity of ATF4, Parkin, PINK1, and LC3B protein expression normalized to β-actin was analyzed by ImageJ software. (**G**) Whole cell lysates extracted from Huh7 cells with empty vector (Mock) and those with HBV construct for 48 h were analyzed by Western blotting with antibodies specific to the indicated proteins.

When mitochondria are depolarized, PTEN-induced putative kinase 1 (PINK1), a mitochondrial Ser/Thr kinase, accumulates on the outer mitochondrial membrane and recruits Parkin to the mitochondria [18]. Parkin translocation to depolarized mitochondria is a hallmark of mitophagy [18], [32], [33]. Thus, we examined mitochondrial translocation of Parkin in HBV-expressing cells by confocal microscopy. Significant Parkin translocation to mitochondria was observed in HBV-expressing cells, compared to untransfected cells (Figure 2A, see merged yellow spots). Similar results were also observed in HBx-expressing cells (Figures S1A and B). Parkin is an E3 ubiquitin ligase which ubiquitinates itself and its mitochondrial substrates, Mfn2 and voltage-dependent anion-selective channel 1 (VDAC1) [18]. Mfn2 functions to promote mitochondrial fusion and its degradation by HBV will favor fission activities [34]. Using purified mitochondrial and cytosolic fractions of HBV-expressing cells, we further analyzed mitochondrial translocation of Parkin. As shown in Figure 2B, Parkin is accumulated and mostly ubiquitinated in the mitochondrial fraction. Parkin ubiquitination was further analyzed by immunoprecipitation with anti-Parkin antibody, followed by subsequent Western blotting with anti-ubiquitin antibody (Figure 2C). We also observed a moderate decrease in the expression levels of VDAC1 (Figure 2B, lanes 2 and 6), whereas Mfn2 is significantly degraded with a concomitant increase in its ubiquitinated form (Figure 2D, first and third panels, lane 3). Parkin-dependent ubiquitination of Mfn2 in HBV-expressing cells was verified by immunoprecipitation assay using HBV-expressing cells with Parkin knockdown (Figure 2D, upper panel, lane 4). These results indicate that HBV stimulates Parkin translocation to mitochondria and Parkin-dependent degradation of Mfn2 by ubiquitination.

### HBV stimulates Parkin, PINK1, and LC3B gene expression

We then investigated whether HBV stimulates the expression of mitophagy-related genes. As shown in Figures 2E and F, HBV gene expression modestly stimulated the expression of Parkin, PINK1, and microtubule-associated protein 1 light chain 3B (LC3B) at both the mRNA and protein levels in HepAD38 cells, but not in HepG2 cells, the parental cell line of HepAD38. An increase in LC3B-I and LC3B-II was also observed (Figure 2F). These results were further confirmed in Huh7 cells transiently expressing HBV genome (Figure 2G). Activating transcription factor 4 (ATF4) was also stimulated by HBV. ATF4 is a transcriptional factor that stimulates Parkin gene expression via unfolded protein response (UPR) [35] (Figure 2F). HBx-expressing cells showed a similar stimulation of Parkin, LC3B-I, and LC3B-II (Figure S1C). By coimmunoprecipitation assay using whole cell lysates extracted from Huh7 cells co-expressing Flag-HBx and mCherry-Parkin protein, physical interaction between HBx and Parkin is shown in Figure S1D. HBx-VDAC3 interaction has been previously reported [4]. Since Parkin binds VDAC [36], it is tempting to speculate that Parkin, VDAC, and HBx may form a ternary complex to expedite the process of Parkin recruitment to mitochondria. In summary, these results demonstrate that HBV/HBx stimulated the expression of mitophagy-related genes.

### HBV/HBx promotes mitophagosome formation associated with Parkin

**Figure 3:**
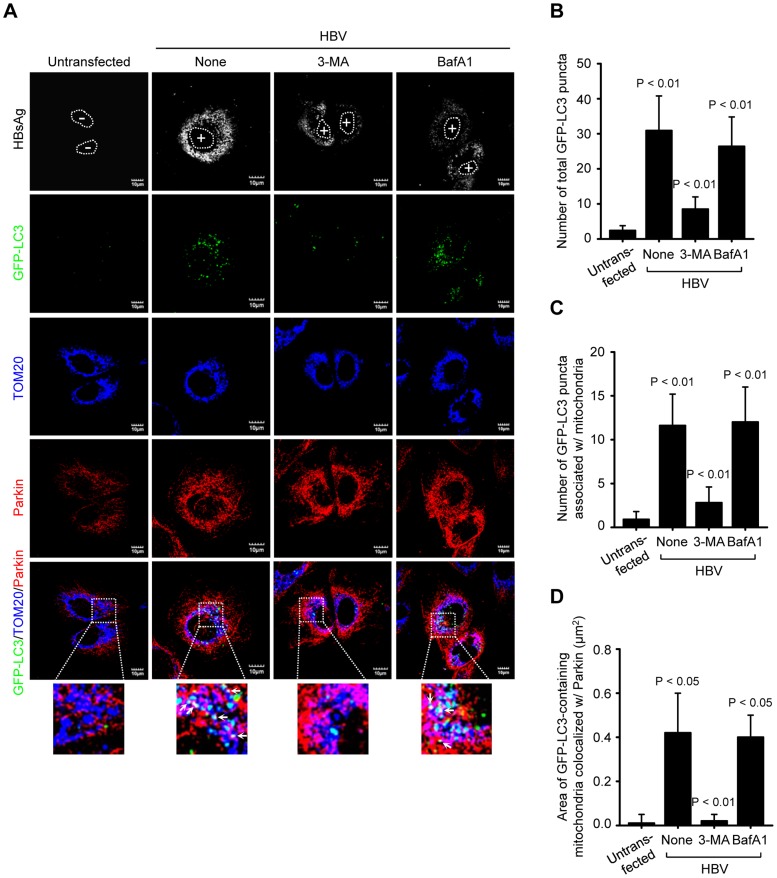
HBV induces mitophagosomes. (**A**) Huh7 cells transiently expressing GFP-LC3 protein were transfected with HBV construct in the absence or presence of 3-MA (10 mM) and BafA1 (100 nM), respectively, for 8 h before fixation. At 2 days post-transfection, cells were immunostained with antibodies against HBsAg (white), TOM20 (blue), and Parkin (red). Nuclei are demarcated with white dots. Transfected (+) and untransfected (−) cells are marked. In the zoomed images, the arrows (white puncta) indicate GFP-LC3 puncta (green) colocalized with TOM20 and Parkin. (**B** and **C**) Quantification of the number of total GFP-LC3 puncta (**B**) and GFP-LC3 puncta colocalized with TOM20 (**C**) in the panel (**A**). (**D**) Quantitative analysis of the area of GFP-LC3 puncta (white) representing merge of GFP-LC3 puncta, TOM20, and Parkin in the panel (**A**).

Next, we analyzed the formation of mitophagosome in Huh7 cells co-expressing HBV and GFP-LC3 protein by confocal microscopy, as we demonstrated the HBV-induced mitochondrial fission and stimulation of Parkin and LC3B expression in Huh7 cells (Figures 1 and 2G). As shown in Figure 3A, we observed that Parkin-containing mitochondria are associated with GFP-LC3 puncta in HBV-expressing cells (see white puncta in the zoomed images). These GFP-LC3 puncta were abrogated upon treatment of cells with autophagy inhibitor 3-methyladenine (3-MA), but not Bafilomycin A1 (BafA1) (Figure 3A). 3-MA inhibits autophagosome formation, whereas BafA1 inhibits fusion of autophagosomes with lysosomes [37], [38]. Quantitative analysis of these results is presented in Figures 3B, C, and D. HBx alone was also capable of forming Parkin-associated mitophagosome (Figure S2A). In contrast, cells expressing whole HBV genome defective in HBx expression (HBV-ΔX) failed to show any GFP-LC3 puncta containing mitochondria, (Figure S2). Quantitative analysis of these images is also presented in Figures S2C, D, and E. Together, these results indicate that HBV/HBx induces Parkin-dependent mitophagosome formation.

### HBV/HBx induces complete mitophagy

**Figure 4:**
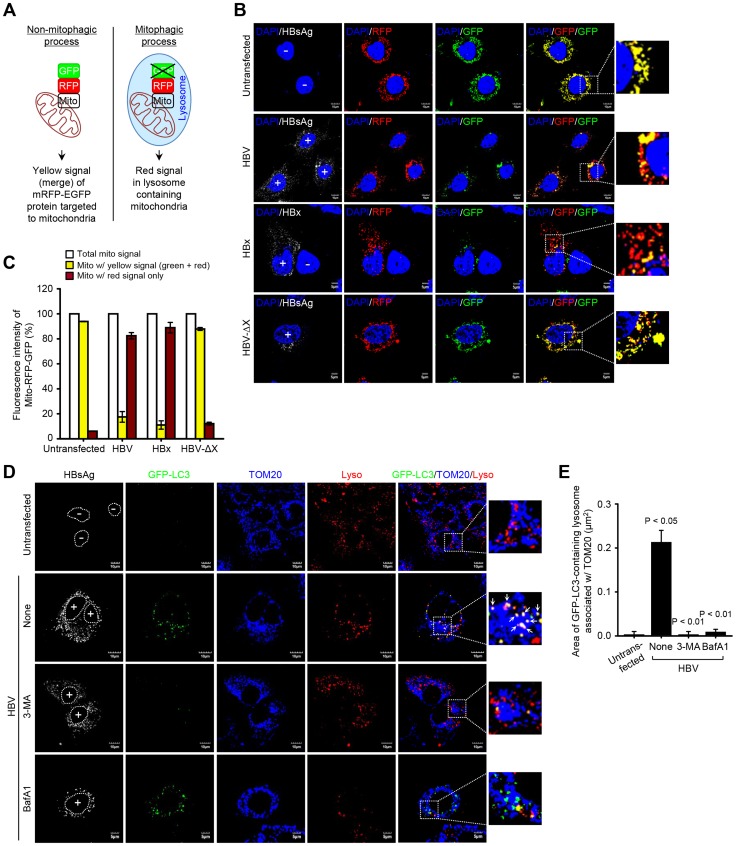
HBV/HBx induces mitophagolysosomes. (**A**) Novel system for monitoring mitophagic flux using dual fluorescence p-mito-mRFP-EGFP reporter (pAT016). Lysosomal delivery of the tandem fusion protein Mito-mRFP-EGFP along with entire mitochondria results in differential quenching and degradation of the two individual fluorochromes, thus allowing for visual analysis of mitophagic flux. (**B**) Huh7 cells transiently expressing Mito-mRFP-EGFP were transfected with HBV, HBx-flag, and HBV-ΔX constructs, respectively, for 48 h. Cells were immunostained with antibodies specific to HBsAg and flag (white), respectively. Nuclei were stained with DAPI (blue). Transfected (+) and untransfected (−) cells are marked. In the zoomed images, fluorescence signals indicate the expression of Mito-mRFP-EGFP targeting mitochondria: yellow color, no mitophagy; red color, mitophagy. (**C**) Quantitative analysis of the fluorescence signal targeted to mitochondria in the panel (**B**). (**D**) GFP-LC3-expressing Huh7 cells were transfected with HBV construct in the absence or presence of 3-MA (10 mM) and BafA1 (100 nM), respectively, for 8 h before fixation. At 2 days post-transfection, cells prestained with LysoTracker (Lyso, red) were immunostained with antibodies against HBsAg (white) and TOM20 (blue). Nuclei are demarcated with white dot circles. Transfected (+) and untransfected (−) cells are marked. In the zoom images, the arrows denoting white puncta indicate GFP-LC3 puncta (green) colocalized with TOM20 and lysosome. (**E**) Quantification of the colocalization of GFP-LC3 puncta containing lysosome with mitochondria in the panel (**D**).

The final step of mitophagy is the fusion of mitophagosomes with lysosomes where the cargo is delivered, degraded and recycled [18]. To analyze the progression of mitophagy from mitophagosomes to lysosomes, we made use of a tandem-tagged RFP-EGFP chimeric plasmid pAT016 encoding a mitochondrial targeting signal sequences fused in-frame with RFP and EGFP genes (Figure 4A), which exploits the differential stabilities of RFP and GFP [39]. GFP signal is quenched in lower pH, while RFP can be visualized in both mitophagosomes and acidic mitophagolysosomes thus the prevalence of RFP fluorescence in the lysosomes indicates completion of mitophagic process. In contrast, under normal conditions, yellow structures indicating merged images of GFP and RFP that localize to mitochondria are observed (Figure 4A). Cells cotransfected with plasmid pAT016 (mito-mRFP-EGFP) and whole HBV genome or, HBV-ΔX, or HBx-flag expressing plasmid, respectively, were analyzed by confocal microscopy. In control cells not expressing HBV or expressing HBV-ΔX, the yellow merged fluorescence that indicates the presence of both EGFP and mRFP in mitochondria was observed (Figure 4B). In contrast, the cells expressing whole HBV genome or HBx displayed distinct red puncta, and fewer green puncta because mRFP is more stable in the lysosomes [39]. When EGFP and mRFP signals are merged, only red puncta were observed in mitophagolysosomes as EGFP signal is quenched in lower pH (Figure 4B). Quantitation of these puncta is presented in Figure 4C. We continued to examine HBV/HBx-induced mitophagolysosome formation by conventional confocal microscopy. As shown in Figures 4D and S3A, GFP-LC3 puncta-containing mitochondria associated with lysosomes were observed in both HBV- and HBx-expressing cells (see white puncta indicating merged images of GFP-LC3, TOM20, and LysoTracker). Treatment with 3-MA and BafA1 abrogated these puncta (Figures 4D and S3A). Quantitative analysis of these results is shown in Figures 4E and S3B, respectively. Collectively, these results demonstrate that HBV and HBx, either expressed alone or in the context of whole HBV genome, respectively, induce complete mitophagy.

As damaged mitochondria are eliminated via mitophagy, we observed a decline in mitochondrial number in HBV-expressing cells, but not in those treated with BafA1 or untransfected cells (Figures S3C and D). Parkin knockdown in HBV-expressing cells also abrogated the decline in mitochondrial number (Figures S3E and F). It should be emphasized that not all mitochondria in HBV/HBx-expressing cells engage in mitophagy. The fraction that undergoes mitophagy and fission is likely to impact the disease process.

### HBV attenuates mitochondrial apoptosis

**Figure 5:**
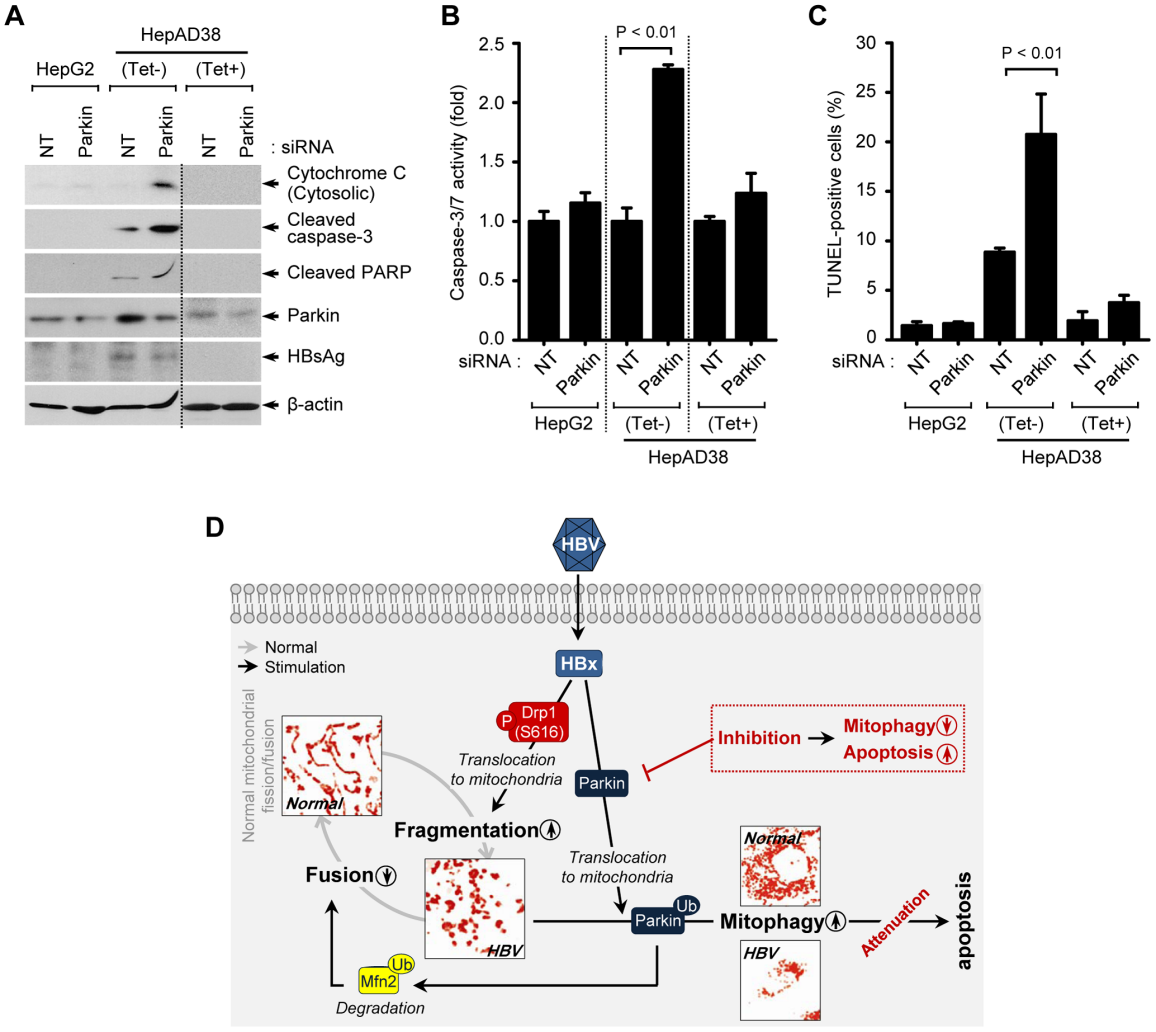
Attenuation of mitochondrial apoptosis by HBV. (**A–C**) Parkin silencing accelerates HBV-induced mitochondrial apoptotic signaling. HepG2 and HepAD38 cells grown in the absence or presence of tetracycline were transfected with non-targeting (NT) and Parkin siRNA, respectively, for 72 h. Cells were used for Western blot analysis using antibodies specific to the indicated proteins (**A**), caspase-3/7 activity assay (**B**), and TUNEL assay (**C**). (**D**) A model elucidating HBV-induced aberrant mitochondrial dynamics in mitochondrial apoptosis. HBV induces immoderate stimulation in mitochondrial fission and mitophagy through mitochondrial translocation of Drp1 and Parkin, respectively, eventually causing disruption in mitochondrial dynamics. Inhibition of mitophagy by Parkin silencing can lead to apoptotic cell death in HBV-infected host cells via accumulation of apoptotic signal (red).

Mitochondrial dynamics is integrally linked to apoptosis [40], [41]. We investigated if HBV enhances mitochondrial fission and mitophagy to modulate apoptotic cell death associated with mitochondrial injury. HepAD38 cells were transfected with Parkin-specific siRNA pool and analyzed for changes in mitochondrial apoptotic signaling pathway. Parkin silencing induced massive cytochrome C release from mitochondria to cytosol and promoted cleavage of poly(ADP-Ribose) polymerase (PARP) and caspase-3, activation of caspase-3/7, and prompted apoptosis as seen by TUNEL assay (Figures 5A–C). These results strongly suggest that HBV-induced mitochondrial dynamics protects virus-infected hepatocytes from apoptotic cell death to facilitate the persistent virus infection (Figure 5D).

## Discussion

Mitochondria are dynamic organelles that undergo fission (fragmented mitochondria), fusion (tubular mitochondrial network), and trafficking [18]. The rapid modulation of mitochondrial dynamics occurs in response to physiological stress, apoptotic stimuli, metabolic demands, and infections [42]. Perturbation in mitochondrial dynamics is involved in many diseases such as neurodegenerative disorders and cardiovascular diseases, underscoring the pivotal importance of this process in maintenance of mitochondrial and cellular homeostasis [42]. Clearance of damaged mitochondria is proposed is orchestrated by asymmetric mitochondrial fission and subsequent elimination of damaged mitochondrial pool by selective mitochondria autophagy (mitophagy) [18]. Fragmented mitochondria are better substrates for removal by mitophagy and elongated or fused mitochondria resist mitophagic degradation, suggesting that mitochondrial dynamics and mitophagy are two critical arms required for maintenance of mitochondrial homeostasis [18], [43]. HBV gene expression is associated with physiological aberrations such as perturbed calcium homeostasis and elevated ROS levels which promote mitochondrial dysfunction and damage [3], [6], [8]. We observed that HBV/HBx expression triggers sequential cascade of events with mitochondria, initiating with their perinuclear accumulation followed by mitochondrial fission and ultimately the clearance of damaged mitochondria by mitophagy. Clearance of damaged mitochondria via mitophagy is crucial for establishing mitochondrial homeostasis and cell survival [18], [43]. Impairment of this process by mutations in mitophagy-mediating genes such PINK1 or Parkin is linked to hereditary forms of Parkinson's disease, a neurodegenerative disorder, emphasizing the vital role of mitochondrial homeostasis in cell survival [44], [45].

Many DNA and RNA viruses have been shown to tightly modulate the autophagy process to prevent their clearance by autophagy, to inhibit host immune response, or to favor viral replication and maturation events [46]. Hepatitis C virus, a positive-sense single-stranded RNA virus, induces Parkin-mediated selective mitophagy which may benefit viral replication [32]. Recently, several reports have described that HBV promotes bulk autophagy to favor its own replication [20]–[24]. Although the molecular mechanisms responsible for induction of autophagy during HBV infection are unclear, it seems that autophagy can either enhance HBV DNA replication or favor HBV envelopment [20]–[24]. HBV may promote autophagy either directly via viral factors that trigger autophagy, or indirectly by mechanisms mediated by virus-induced physiological aberrations and stress. Here, our data suggests a functional crosstalk between virus and autophagy pathways providing evidence that selective, rather than bulk autophagy is probably involved in promoting the viability of HBV-infected hepatocytes from imminent cell death due to the virus-mediated mitochondrial injury. This is in line with the emerging concept that selective autophagy of organelles contributes to tight control of host-pathogen interactions [46].

Although the role of HBV and HBx in regulating apoptotic signaling has long been debated [47], [48], based on the *in vivo* studies, it is inferred that HBV-infected hepatocytes maintain a persistent phenotype and that HBV replicates within infected hepatocytes noncytopathically [49]. The noncytopathic viruses have evolved mechanisms to mitigate the adverse effects of the pathophysiological perturbations manifested in the host cells due to recurrent infection. Here, we demonstrate that HBV modulates mitochondrial dynamics to promote mitochondrial fission and subsequent clearance of damaged mitochondria by mitophagy. Damaged mitochondria are a major source of ROS and can trigger a continuous and vicious cycle of subsequent damage to healthy mitochondria followed by ROS generation, ultimately leading to cell death [17]. Hence, a rapid turnover and clearance of damaged mitochondria is needed to confound imminent cell death due to mitochondrial injury accrued during HBV infection and sustain the viability of the infected cells. In agreement with this assumption, we observed a surge in mitochondrial-apoptotic signaling and resultant death of the HBV-infected hepatocytes upon inhibition of the mitophagy pathway. Hepatocyte damage commonly observed during chronic hepatitis is widely believed to be immune-mediated [49] and the failure to mount an efficient immune response to eliminate the infected hepatocytes is considered the primary cause for persistence of chronic infections, including HBV. However the intracellular mechanism(s), which prevents cell death and support the viability of infected cells in conditions of virus-induced adverse intracellular physiology contributes to the lack of virus-induced cytopathology in non-lytic viruses and probably play a significant role in persistence of non-lytic chronic infections. However, further studies are required to confirm the significance of our findings in *in vivo* conditions. Currently, the most convenient way to test our hypothesis *in vivo* settings is the use of animal models of HBV infection such as Woodchucks, Ducks, or Tupaia to determine if abrogation of Parkin-mediated mitophagy pathway promotes specific death of HBV-infected hepatocytes and alleviates persistent HBV infection in these animals.

It is believed that HBx sensitizes and indirectly participates in the onset of liver oncogenesis [3], [47]. Its pleiotropic functions in activating host gene expression, interaction with numerous cellular targets, and its imminent role in altering mitochondrial physiology, promoting oxidative stress, and affecting the epigenetic changes in the chromatin collectively influence the early steps of liver neoplasia. Although not directly linked, HBx-mediated mitochondrial damage and aberrant mitochondrial dynamic may also contribute in pathogenesis of liver disease and HCC [50], [51].

In summary, this study provides a unique insight into the probable involvement of HBV-induced altered mitochondrial dynamics and mitophagy in possibly facilitating the persistence of infection and pathogenesis of liver disease associated with infection thus unraveling potential newer avenues for design of novel therapeutics against chronic HBV infection.

## Materials and Methods

### Cell culture

Human hepatoma cell line Huh7 was grown in high-glucose DMEM (Gibco) supplemented with 10% fetal bovine serum (Hyclone), 1% MEM non-essential amino acid (Gibco), 100 units/ml penicillin (Gibco), and 100 µg/ml streptomycin (Gibco). Human hepatoma HepG2 and HepAD38 cells harboring HBV full-length genome were maintained in RPMI 1640 (Gibco) supplemented with 20% fetal bovine serum, 1% MEM non-essential amino acid, 100 units/ml penicillin, and 100 µg/ml streptomycin. In addition, HepAD38 cells were grown in the presence of 0.5 mg/ml G418 (Invitrogen) and 1 µg/ml tetracycline. NT-KD (expressing non-target shRNA) and P-KD (expressing Parkin shRNA) cells used in this study were maintained in the presence of 2.5 µg/ml of puromycin, as described previously and knockdown level of Parkin gene was shown in the previous study [32].

### DNA constructs

The pHBV1.3mer and pHBV-ΔX plasmid DNAs encoding wild-type HBV genome and HBx-deficient HBV genome, respectively, were a kind gift from Dr. Jing-hsiung James Ou (University of Southern California). The pHBx-flag plasmid DNA was described previously [4]. The pEGFP-LC3 plasmid DNA was a kind gift from Dr. Tamotsu Yoshimori (National Institute of Genetics, Japan). The mCherry-Parkin plasmid DNA (plasmid #23956) was obtained from Addgene (a generous gift of Dr. Richard Youle, National Institute of Health, Bethesda, MD). HepAd38 cells were a kind gift of Dr. Christoph Seeger (Fox Chase Cancer Center, Philadelphia, PA). To create plasmid pAT016 (p-mito-mRFP-EGFP), plasmid ptfLC3 (Addgene plasmid #2174, a generous gift of Dr. Tamotsu Yoshimori) was double digested with *Bgl*2 and *Bam*HI to remove LC3 coding sequences, resulting in plasmid p-mRFP-EGFP production and then, polymerase chain reaction product for mitochondrial targeting signal sequences of human cytochrome c oxidase subunit VIII amplified from pEYFP-mito (Clontech) was inserted N-terminally in frame into p-mRFP-EGFP.

### Immunofluorescence

To conduct laser scanning confocal microscopy, the cells grown on coverslips were transfected with the indicated plasmid DNAs followed by immunofluorescence assay, as described previously [32]. Images were visualized under a 60× or 100× oil objectives using an Olympus FluoView 1000 confocal microscope. Quantification of images (at least 10 cells per each sample) was conducted with ImageJ and MBF ImageJ softwares.

### Reagents and antibodies

Chemical reagents used in this study were Bafilomycin A1 (Enzo Life Sciences) and 3-Methyladenine (Sigma). Primary antibodies used in this study include the following: rabbit monoclonal anti-Drp1 (Cell Signaling); rabbit monoclonal anti-phospho-Drp1 (S616) (Cell Signaling); rabbit monoclonal anti-ATF4 (Cell Signaling); rabbit polyclonal anti-Parkin (Abcam); rabbit monoclonal anti-LC3B (Cell Signaling); rabbit polyclonal anti-PINK1 (Abcam); rabbit polyclonal anti-VDAC1 (Cell Signaling); mouse monoclonal anti-Mfn2 (Abcam); goat polyclonal anti-VDAC3 (Santa Cruz); rabbit polyclonal anti-GAPDH (Santa Cruz); goat polyclonal anti-β-actin (Santa Cruz); mouse monoclonal anti-TOM20 (BD); rabbit polyclonal anti-TOM20 (Abcam); mouse monoclonal anti-Flag M2 (Sigma); rabbit polyclonal anti-DTKDDDDK-tag (GenScript); goat polyclonal anti-DDDDK (Abcam); mouse monoclonal anti-HBsAg (Thermo Scientific); mouse monoclonal anti-Ubiquitin (Cell Signaling); rabbit monoclonal anti-cleaved PARP (Cell Signaling); rabbit monoclonal anti-cleaved caspase-3 (Cell Signaling); rabbit polyclonal anti-cytochrome c (Cell Signaling); normal rabbit IgG (Cell Signaling); normal mouse IgG (Santa Cruz). The secondary antibodies used for immunofluorescence were Alexa Fluor 350, 488, 594, or 647 donkey anti-mouse, rabbit, or goat IgG (Molecular Probe). The secondary antibodies used for Western blot analysis were HRP-conjugated anti-mouse IgG (Cell Signaling), HRP-conjugated anti-rabbit IgG (Cell Signaling), and HRP-conjugated anti-goat IgG (Jackson Laboratories).

### siRNA transfection

Small interfering RNA (siRNA) pools used in this study were siGENOME SMARTpool for Parkin (NM_004562) and non-targeting #1 control (NT) (Dharmacon). The cells were transfected with siRNA (50 nM) for the indicated times using DharmaFECT 4 transfection reagent according to the manufacturer's instructions (Dharmacon).

### Real-time qRT-PCR

To analyze the expression levels of Parkin, PINK1, and LC3B genes, total cellular RNA and subsequent complementary DNAs were prepared, as described previously [32]. The RNA levels of Parkin, PINK1, and LC3B were quantified by real-time qRT-PCR using DyNAmo HS SYBR Green qPCR kit (Finnzymes). The following primer sets were used for RT-PCR: Parkin forward, 5′-TACGTGCACAGACGTCAGGAG; Parkin reverse, 5′-GACAGCCAGCCACACAAGGC; PINK1 forward, 5′-GGGGAGTATGGAGCAGTCAC; PINK1 reverse, 5′-CATCAGGGTAGTCGACCAGG; LC3B forward, 5′- GAGAAGACCTTCAAGCAGCG; LC3B reverse, 5′- AAGCTGCTTCTCACCCTTGT; GAPDH forward, 5′-GCCATCAATGACCCCTTCATT; and GAPDH reverse, 5′-TTGACGGTGCCATGGAATTT. Real-time qPCR was conducted by using an ABI PRISM 7000 Sequence Detection System (Applied Biosystems).

### Immunoprecipitation and Western blot analysis

For Western blot analysis, whole cell lysates (WCL) were extracted from cells, subjected to SDS-PAGE, transferred to nitrocellulose membrane (Thermo Scientific), and Western blot analyzed with antibodies against the indicated proteins, as described previously [32]. For analysis of ubiquitinated Parkin and Mfn2 in WCL, immunoprecipitates were prepared followed by Western blotting with anti-ubiquitin antibody, as described previously [32]. For co-immunoprecipitation, Huh7 cells co-transfected with HBx-flag and mCherry-Parkin were suspended in 0.1 ml of RIPA buffer. The suspended cells were incubated for 20 min on ice and clarified by centrifugation at 15,000×g at 4°C for 20 min. The supernatant was mixed with 1.9 ml of RIPA buffer without SDS and immunoprecipitated with anti-flag antibody and protein-G Sepharose. The immunoprecipitates were Western blot analyzed with anti-Parkin, flag, and VDAC3 antibodies, respectively. The intensity of protein expression was quantified by ImageJ software.

### Subcellular fractionation

To isolate pure cytosolic and mitochondrial fraction, HepAD38 cells were homogenized and isolated, as described previously [32], [52].

### Caspase-3/7 assay

The activity of caspase-3/7 in HepG2 and HepAD38 cells transfected with siRNA were measured by using Caspase-Glo 3/7 assay kit according to the manufacturer's instructions (Promega).

### TUNEL assay

Apoptotic cells death in HepG2 and HepAD38 cells transfected with siRNA were measured by using Click-iT TUNEL Alexa Fluor 488 imaging assay kit according to the manufacturer's instructions (Invitrogen). For quantitative analysis, at least 1,000 cells on immunofluorescence image were counted.

### Statistical analysis

Statistical analyses using Student's t-test were performed by using Sigma Plot software (Systat Software Inc., San Jose, CA, USA).

## Supporting Information

Figure S1 **HBx stimulates Parkin gene expression, triggers Parkin recruitment to mitochondria, and physically interacts with Parkin and VDAC3.** (**A**) Confocal microscopy of Parkin aggregates on the mitochondria in HBx-expressing cells. Huh7 cells transfected with HBx-flag construct were prestained with MitoTracker (Mito, red) and immunostained with anti-Parkin (green) and anti-flag (white) antibodies. Nuclei are demarcated with white dot circles. Transfected (+) and untransfected (−) cells are marked. The zoom images display the accumulation of endogenous Parkin on the mitochondria. (**B**) Quantification of fluorescence intensity of Parkin aggregates on the mitochondria (mean ± SEM; n≥10 cells; *p<0.001). (**C**) Whole cell lysates extracted from Huh7 cells transfected with HBx-flag construct for 48 h were analyzed by Western blotting with antibodies specific for the indicated proteins. (**D**) HBx protein in whole cell lysates extracted from Huh7 cells co-transfected with HBx-flag and mCherry-Parkin constructs was immunoprecipitated by anti-flag antibody, followed by Western blotting with antibodies specific for the indicated proteins. Normal mouse IgG was used as a negative control (Ctrl Ab) for co-immunoprecipitation (co-IP).
(PDF)

Figure S2 **HBx induces Parkin-mediated mitophagosome formation.** (**A**) Huh7 cells transiently expressing GFP-LC3 protein were transfected with HBx-flag construct in the absence or presence of 3-MA (10 mM) and BafA1 (100 nM), respectively, for 8 h before fixation. At 2 days post-transfection, cells were immunostained with antibodies specific to flag (white), TOM20 (blue), and Parkin (red). (**B**) GFP-LC3-expressing Huh7 cells were transfected with HBV-ΔX construct. At 36 hours post-transfection, cells prestained with MitoTracker (Mito, red) were immunostained with anti-Parkin (green) and anti-HBsAg (white) antibodies. Nuclei are demarcated with white dot circles. Transfected (+) and untransfected (−) cells are marked. (**A** and **B**) The arrows (white puncta) in the zoom images display the merge of GFP-LC3 puncta (green), TOM20 (**A**)/Mito (**B**), and Parkin. (**C** and **D**) Quantification of the number of total GFP-LC3 puncta (**C**) and GFP-LC3 puncta colocalized with TOM20 (**D**) in the panels (**A**) and (**B**) (mean ± SEM; n≥10 cells; *p<0.001). (**E**) Quantitative analysis of the area of GFP-LC3 puncta (white) representing merge of GFP-LC3 puncta, TOM20/Mito, and Parkin in the panels (**A**) and (**B**) (mean ± SEM; n≥10 cells; *p<0.001).
(PDF)

Figure S3 **HBx induces complete mitophagy.** (**A** and **B**) Confocal microscopy showing complete mitophagic process in HBx-expressing cells. (**A**) Huh7 cells transiently expressing GFP-LC3 protein were transfected with HBx-flag DNA construct in the absence or presence of 3-MA (10 mM) and BafA1 (100 nM), respectively, for 8 h before fixation. At 2 days post-transfection, cells prestained with LysoTracker (Lyso, red) were immunostained with anti-flag (white) and TOM20 (blue) antibodies. Nuclei are demarcated with white dot circles. Transfected (+) and untransfected (−) cells are marked. In the zoom images, the arrows (white puncta) indicate GFP-LC3 puncta (green) colocalized with TOM20 and lysosome. (**B**) Quantification of the colocalization of GFP-LC3 puncta containing lysosome with mitochondria in the panel (A) (mean ± SEM; n≥10 cells; *p<0.001). (**C** and **D**) Rescue effect of BafA1 in HBV-induced decline of mitochondria. Huh7 cells transfected with HBV construct were treated BafA1 (50 nM) for 12 h before fixation. At 36 hours post-transfection, cells prestained with MitoTracker (Mito, red) were immunostained with anti-HBsAg antibody (green) (**C** and **E**). (**E** and **F**) Inhibitory effect of Parkin silencing in HBV-induced decline of mitochondria. Stable cells expressing Parkin-specific shRNA (P-KD) were transfected with HBV DNA construct. Stable cells expressing non-targeting shRNA (NT-KD) were used as a negative control. Nuclei are demarcated with white dot circles (**C**) or were stained with DAPI (blue) (**E**). Transfected (+) and untransfected (−) cells are marked. The zoomed images indicate mitochondrial morphology in transfected and untransfected cells, respectively. (**D** and **F**) Quantitative analysis of mitochondria-specific fluorescence intensity in the panels (**C**) and (**E**) (mean ± SEM; n≥10 cells; *p<0.01, **p<0.001).
(PDF)
