# Supplementary figures and images for: Hepatitis B Virus Disrupts Mitochondrial Dynamics: Induces Fission and Mitophagy to Attenuate Apoptosis
Source: PLoS Pathog. 2013 Dec 5;9(12):e1003722. doi: 10.1371/journal.ppat.1003722 (PMC3855539; doi:10.1371/journal.ppat.1003722)

# Figure S1

**A**

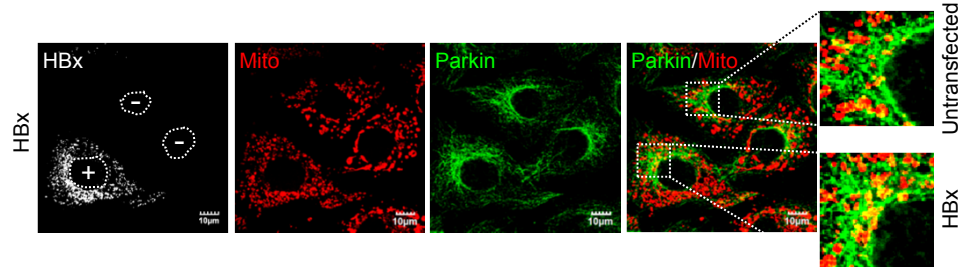

**B**

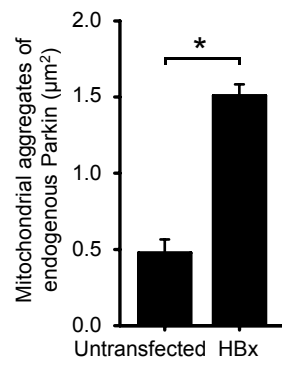

**C**

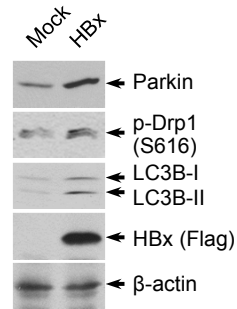

**D**

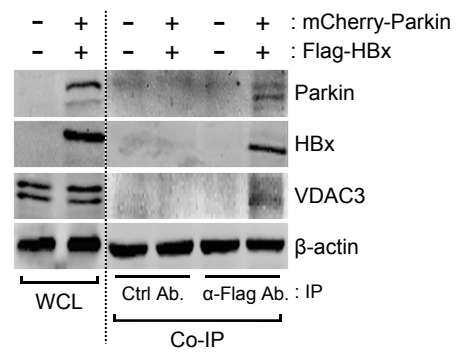

Supplement: Figure S1 — HBx stimulates Parkin gene expression, triggers Parkin recruitment to mitochondria, and physically interacts with Parkin and VDAC3. (A) Confocal microscopy of Parkin aggregates on the mitochondria in HBx-expressing cells. Huh7 cells transfected with HBx-flag construct were prestained with MitoTracker (Mito, red) and immunostained with anti-Parkin (green) and anti-flag (white) antibodies. Nuclei are demarcated with white dot circles. Transfected (+) and untransfected (−) cells are marked. The zoom images display the accumulation of endogenous Parkin on the mitochondria. (B) Quantification of fluorescence intensity of Parkin aggregates on the mitochondria (mean ± SEM; n≥10 cells; *p<0.001). (C) Whole cell lysates extracted from Huh7 cells transfected with HBx-flag construct for 48 h were analyzed by Western blotting with antibodies specific for the indicated proteins. (D) HBx protein in whole cell lysates extracted from Huh7 cells co-transfected with HBx-flag and mCherry-Parkin constructs was immunoprecipitated by anti-flag antibody, followed by Western blotting with antibodies specific for the indicated proteins. Normal mouse IgG was used as a negative control (Ctrl Ab) for co-immunoprecipitation (co-IP). (PDF) [file ppat.1003722.s001.pdf]

# Figure S2

**A**

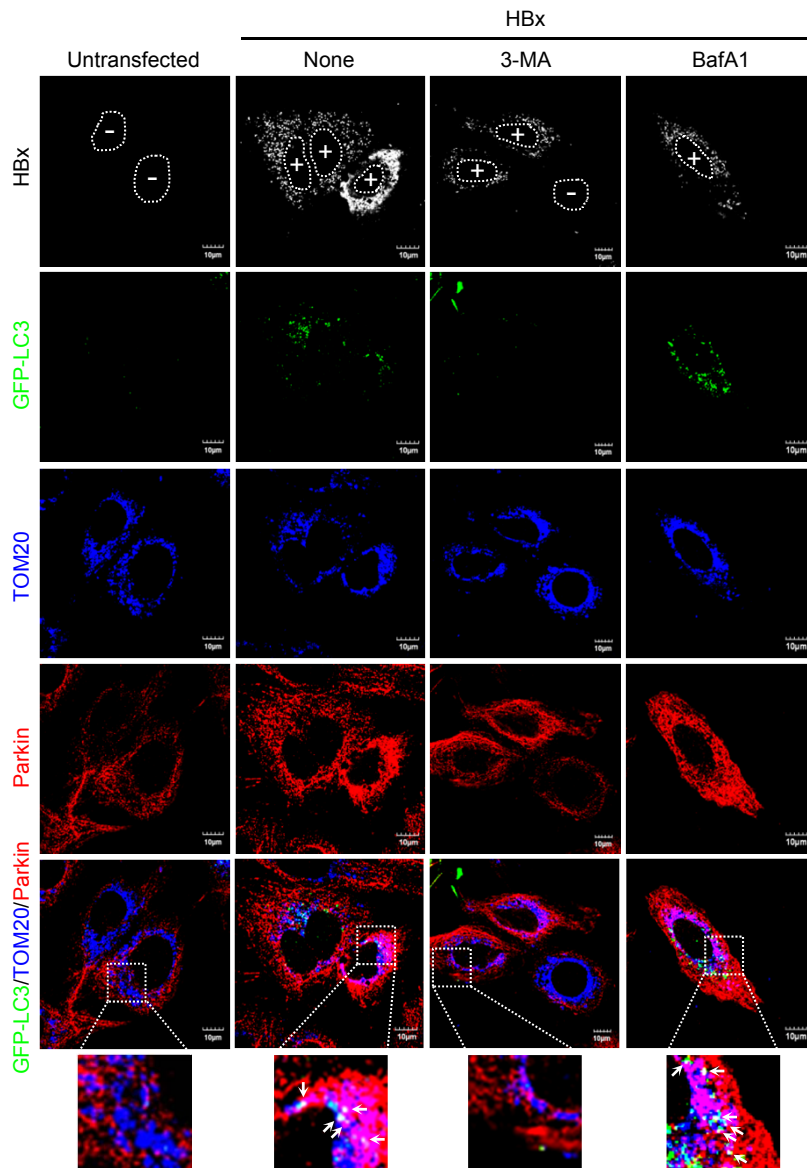

**B**

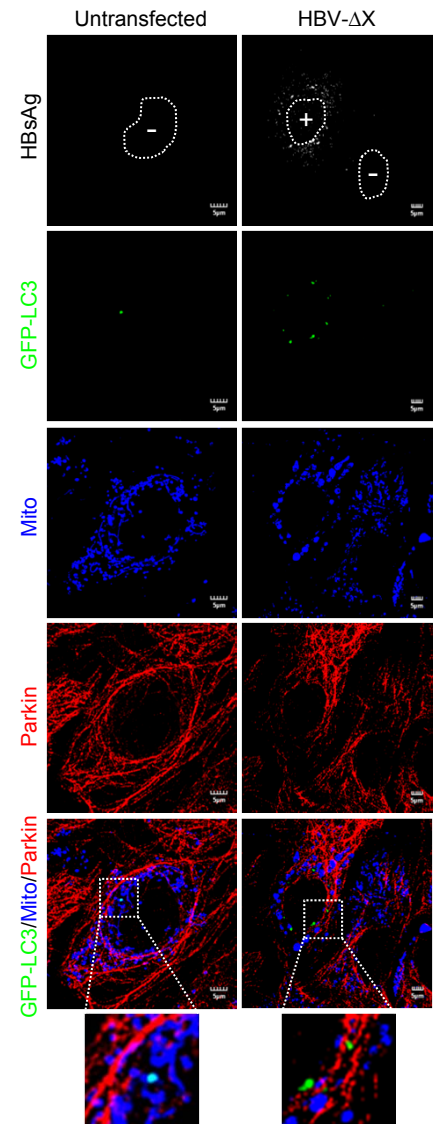

**C**

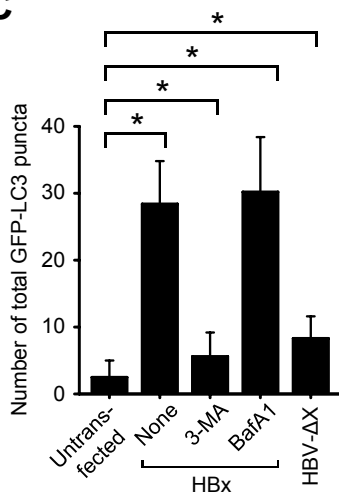

**D**

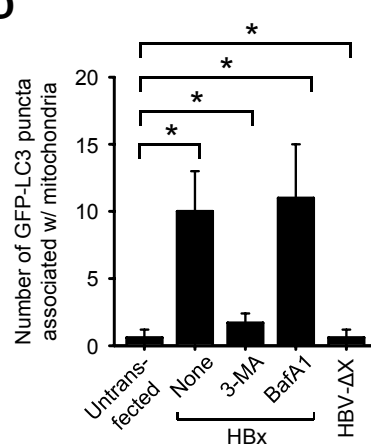

**E**

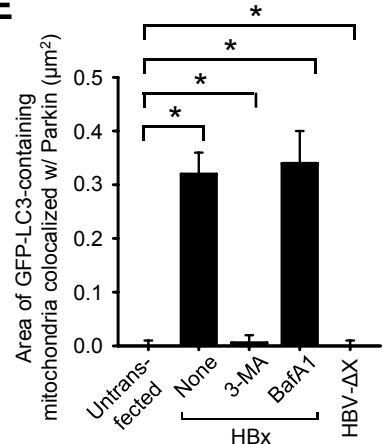

Supplement: Figure S2 — HBx induces Parkin-mediated mitophagosome formation. (A) Huh7 cells transiently expressing GFP-LC3 protein were transfected with HBx-flag construct in the absence or presence of 3-MA (10 mM) and BafA1 (100 nM), respectively, for 8 h before fixation. At 2 days post-transfection, cells were immunostained with antibodies specific to flag (white), TOM20 (blue), and Parkin (red). (B) GFP-LC3-expressing Huh7 cells were transfected with HBV-ΔX construct. At 36 hours post-transfection, cells prestained with MitoTracker (Mito, red) were immunostained with anti-Parkin (green) and anti-HBsAg (white) antibodies. Nuclei are demarcated with white dot circles. Transfected (+) and untransfected (−) cells are marked. (A and B) The arrows (white puncta) in the zoom images display the merge of GFP-LC3 puncta (green), TOM20 (A)/Mito (B), and Parkin. (C and D) Quantification of the number of total GFP-LC3 puncta (C) and GFP-LC3 puncta colocalized with TOM20 (D) in the panels (A) and (B) (mean ± SEM; n≥10 cells; *p<0.001). (E) Quantitative analysis of the area of GFP-LC3 puncta (white) representing merge of GFP-LC3 puncta, TOM20/Mito, and Parkin in the panels (A) and (B) (mean ± SEM; n≥10 cells; *p<0.001). (PDF) [file ppat.1003722.s002.pdf]

# Figure S3

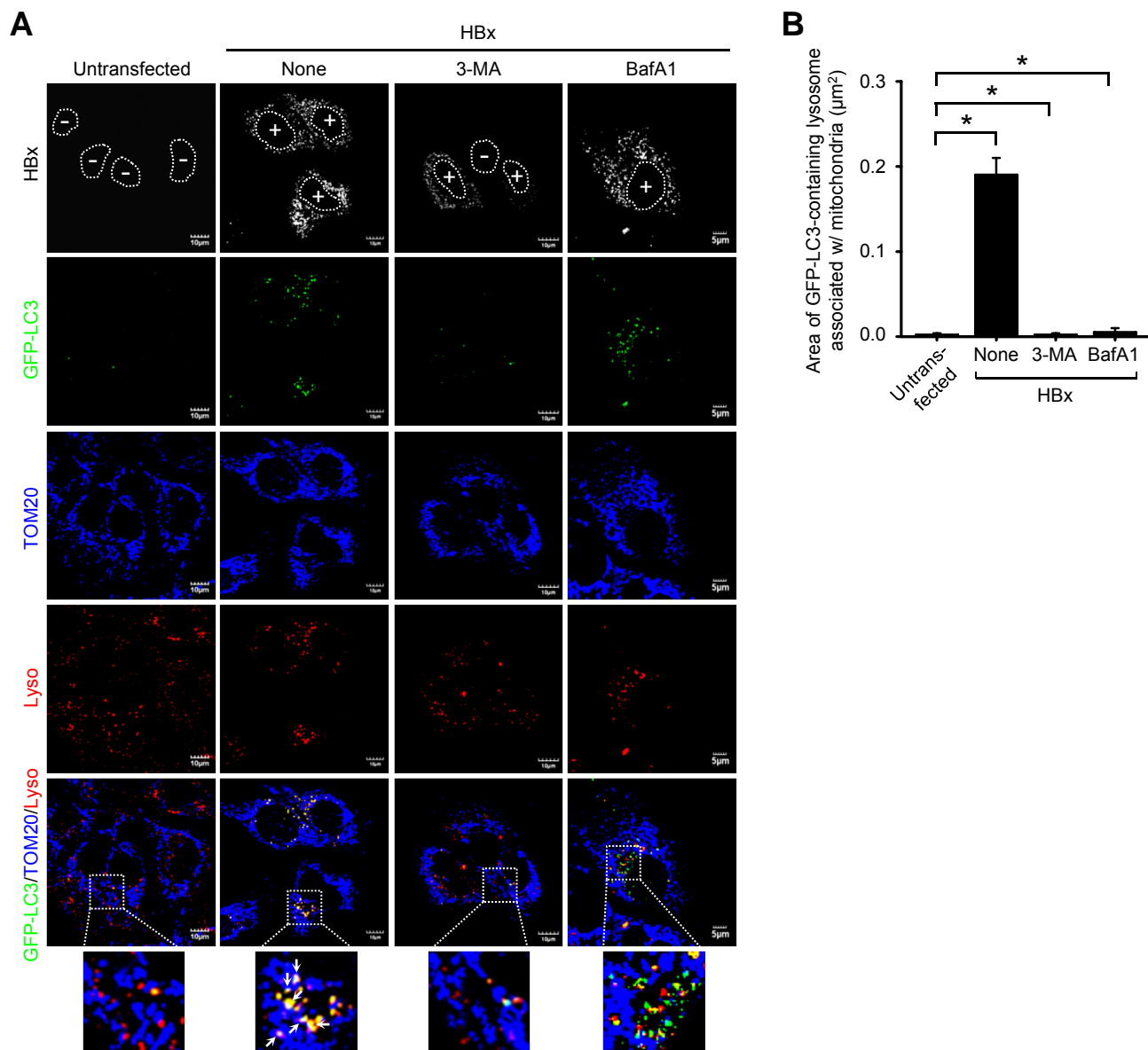

Figure S3 continued

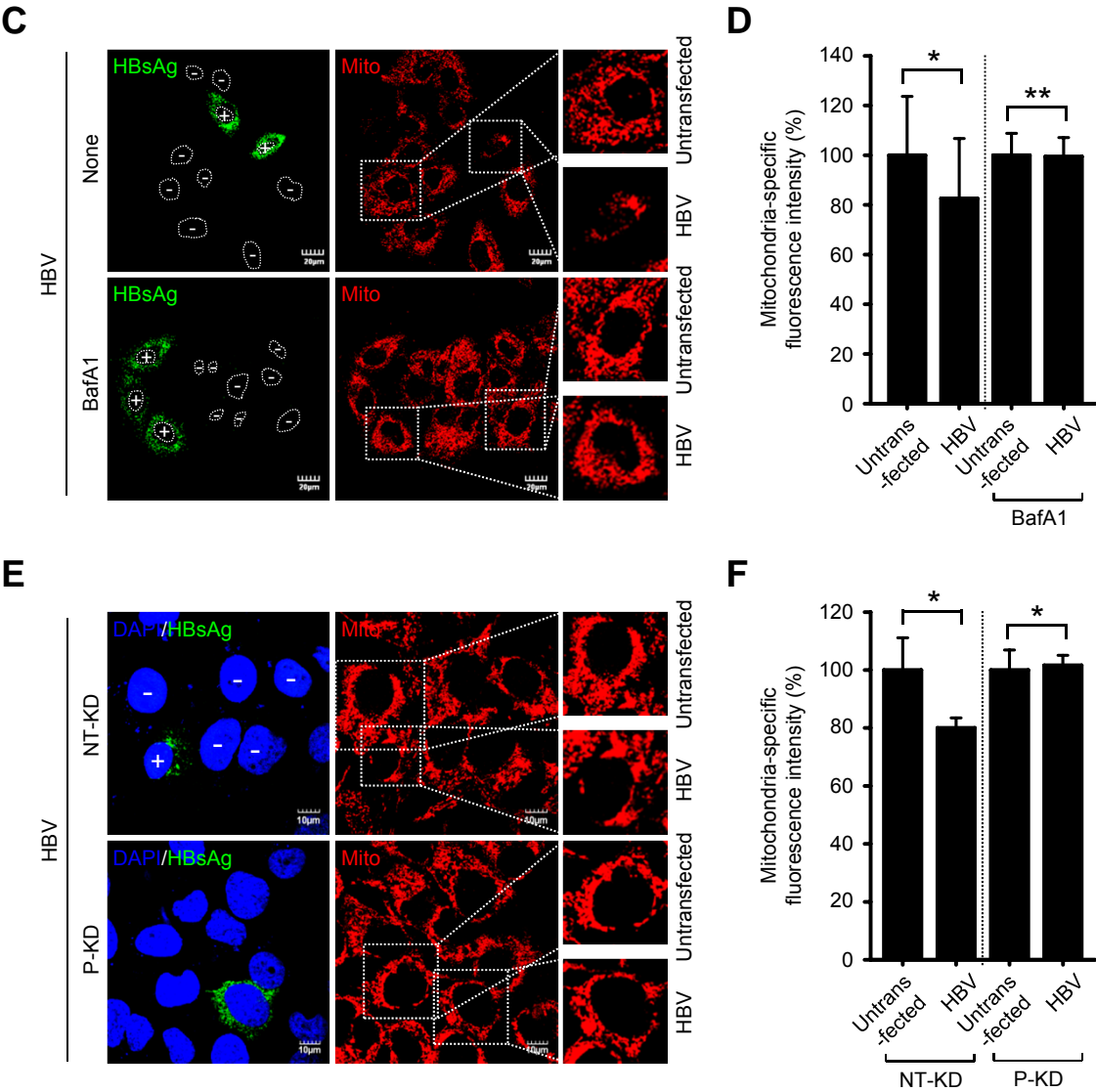

Supplement: Figure S3 — HBx induces complete mitophagy. (A and B) Confocal microscopy showing complete mitophagic process in HBx-expressing cells. (A) Huh7 cells transiently expressing GFP-LC3 protein were transfected with HBx-flag DNA construct in the absence or presence of 3-MA (10 mM) and BafA1 (100 nM), respectively, for 8 h before fixation. At 2 days post-transfection, cells prestained with LysoTracker (Lyso, red) were immunostained with anti-flag (white) and TOM20 (blue) antibodies. Nuclei are demarcated with white dot circles. Transfected (+) and untransfected (−) cells are marked. In the zoom images, the arrows (white puncta) indicate GFP-LC3 puncta (green) colocalized with TOM20 and lysosome. (B) Quantification of the colocalization of GFP-LC3 puncta containing lysosome with mitochondria in the panel (A) (mean ± SEM; n≥10 cells; *p<0.001). (C and D) Rescue effect of BafA1 in HBV-induced decline of mitochondria. Huh7 cells transfected with HBV construct were treated BafA1 (50 nM) for 12 h before fixation. At 36 hours post-transfection, cells prestained with MitoTracker (Mito, red) were immunostained with anti-HBsAg antibody (green) (C and E). (E and F) Inhibitory effect of Parkin silencing in HBV-induced decline of mitochondria. Stable cells expressing Parkin-specific shRNA (P-KD) were transfected with HBV DNA construct. Stable cells expressing non-targeting shRNA (NT-KD) were used as a negative control. Nuclei are demarcated with white dot circles (C) or were stained with DAPI (blue) (E). Transfected (+) and untransfected (−) cells are marked. The zoomed images indicate mitochondrial morphology in transfected and untransfected cells, respectively. (D and F) Quantitative analysis of mitochondria-specific fluorescence intensity in the panels (C) and (E) (mean ± SEM; n≥10 cells; *p<0.01, **p<0.001). (PDF) [file ppat.1003722.s003.pdf]
